# Inhibition of GSK3β Promotes Proliferation and Suppresses Apoptosis of Porcine Muscle Satellite Cells

**DOI:** 10.3390/ani12233328

**Published:** 2022-11-28

**Authors:** Jinryong Park, Hyunwoo Choi, Kwanseob Shim

**Affiliations:** 1 Department of Stem Cell and Regenerative Biotechnology, Konkuk University, Seoul 05029, Republic of Korea; 2 3D Tissue Culture Research Center, Konkuk University, Seoul 05029, Republic of Korea; 3 Department of Animal Science, Jeonbuk National University, Jeonju 54896, Republic of Korea; 4 Department of Agricultural Convergence Technology, Jeonbuk National University, Jeonju 54896, Republic of Korea; 5 Department of Animal Biotechnology, Jeonbuk National University, Jeonju 54896, Republic of Korea

**Keywords:** satellite cell, maintenance, proliferation, Wnt signaling pathway, porcine

## Abstract

**Simple Summary:**

This study was conducted to investigate the effect of the inhibitor and activator of the Wnt signaling pathway on the proliferation and maintenance of satellite cells in vitro. In this study, we isolated porcine muscle satellite cells (PMSCs) from a 1-day-old piglet and cultured PMSCs by treating the inhibitor (XAV939, Tankyrase inhibitor) and activator (CHIR99021, GSK3β inhibitor) of Wnt signaling. The results revealed that CHIR99021 promoted the proliferation of PMSCs by inhibiting MyoD expression while maintaining the expression of Pax7. CHIR99021 also suppressed apoptosis of PMSCs by regulating the expression of apoptosis-related proteins and genes. In essence, our findings indicate that the inhibition of GSK3β could promote the self-renewal of PMSCs and inhibit apoptosis.

**Abstract:**

As the global population increases, interest in cultured meat (a new research field) is gradually increasing. The main raw material for the production of cultured meat is muscle stem cells called satellite cells isolated from livestock. However, how to mass proliferate and maintain satellite cells in vitro without genetic manipulation remains unclear. In the present study, we isolated and purified porcine muscle satellite cells (PMSCs) from the femur of a 1-day-old piglet and cultured PMSCs by treating them with an inhibitor (XAV939, Tankyrase (TNKS) inhibitor) or an activator (CHIR99021, glycogen synthase kinase 3 beta (GSK3β) inhibitor) of Wnt signaling. The CHIR group treated with 3 μM CHIR99021 showed a significantly increased proliferation rate of PMSCs compared to the SC group (control), whereas the XAV group treated with 1 μM XAV939 showed a significantly decreased proliferation rate of PMSCs. CHIR99021 also inhibited the differentiation of PMSCs by reducing the expression of MyoD while maintaining the expression of Pax7 and suppressed apoptosis by regulating the expression of apoptosis-related proteins and genes. RNA sequencing was performed to obtain gene expression profiles following inhibition or activation of the Wnt signaling pathway and various signaling mechanisms related to the maintenance of satellite cells were identified. Our results suggest that inhibition of GSK3β could dramatically improve the maintenance and mass proliferation ability of PMSCs in vitro by regulating the expression of myogenic markers and the cell cycle.

## 1. Introduction

As negative perceptions of traditional meat consumption increase, cultured meat, a field of cellular agriculture using stem cells and tissue engineering technology, is emerging as an alternative [1]. For efficient production of cultured meat, it is important to mass proliferate muscle stem cells in vitro [2]. Among cells that constitute skeletal muscles, there is a population of undifferentiated muscle precursor cells (i.e., muscle stem cells or satellite cells) [3]. These cells exist in a niche between the myofiber membrane and the basal layer. They usually exist in a quiescent state [4]. When the skeletal muscle is stimulated, such as an injury, quiescent satellite cells are activated. They can proliferate (i.e., myoblasts) and proceed to muscle regeneration. Some myoblasts can maintain the pool of satellite cells through self-renewal [5]. However, these characteristics are only maintained when satellite cells are isolated and then immediately transplanted into another individual. When these satellite cells are cultured in vitro, they will lose their stemness [6].

To promote the proliferation of satellite cells and inhibit their differentiation in vitro, many studies were conducted by adding various growth factors and cytokines to the culture medium [7,8,9,10,11]. In addition, various cytokine mechanisms such as TGF-β, Notch, MAPK, PI3K and Wnt signaling were verified to be associated with the control of biological processes in satellite cells [12,13]. Among them, Wnt signaling is essential for the self-renewal of embryonic stem cells (ESCs) and Wnt proteins inhibit differentiation into epiblast stem cells (EpiSCs) [14]. This signaling acts as a crucial regulator of skeletal muscle stem cells and ESCs [15]. Constitutive expression of β-catenin, a main factor in the canonical Wnt signaling pathway, can increase the ratio of pax7^(+)^/MyoD^(−)^ satellite cells and promote self-renewal of skeletal muscle satellite cells [12]. Fleming et al. [16] reported that Wnt signaling contributes to maintaining the quiescent state of hematopoietic stem cells (HSCs). In contrast, there are conflicting studies showing that β-catenin can promote muscle differentiation by interacting with MyoD [17] and that Wnt signaling promotes phylogenetic progression rather than maintains the stemness of stem cells [18].

Although many studies have been carried out on Wnt signaling in various stem cells, most studies were conducted in human- and mouse-derived cells and few studies have verified the effect of Wnt signaling in the porcine muscle that we use as food. We conducted this study to confirm the effect of Wnt signaling regulation on porcine muscle satellite cells in vitro and to apply it to cultured meat production. Here, we investigated the effect of the inhibitor (XAV939) or activator (CHIR99021) of the Wnt signaling pathway on the proliferation and maintenance of porcine muscle satellite cells in vitro. In this study, we showed that inhibition of GSK3β could promote the self-renewal of PMSCs and inhibit their apoptosis. In addition, we discussed the mechanism involved in the regulation of CHIR99021 on satellite cells through KEGG pathway analysis.

## 2. Materials and Methods

### 2.1. Ethical Approval

All animal care and experimental procedures were approved by the Animal Ethics Committee of Jeonbuk National University (JBNU 2020-0147), Republic of Korea. All experiments were carried out in accordance with relevant guidelines and regulations of Jeonbuk National University, and compliant with ARRIVE guidelines.

### 2.2. Porcine Muscle Satellite Cell (PMSC) Derivation and Culture

Muscle tissues were derived from the femur of 1-day-old piglets and immediately washed three times with Dulbecco`s phosphate buffer saline (DPBS) (Gibco, Carlsbad, CA, USA, #14190–144) containing 10% Penicillin–streptomycin (PS) (Gibco, Carlsbad, CA, USA, #15140–122). Muscle tissues were chopped into tiny pieces and dissociated with a digestive solution containing collagenase D (Roche, Indianapolis, IN, USA, #11088858001, 2 mg/mL), dispase II (Roche, Indianapolis, IN, USA, #4942078001, 1 U/mL), and 0.25% trypsin-EDTA (Gibco, Carlsbad, CA, USA, #25200–072) in DMEM/F12 (Gibco, Carlsbad, CA, USA, #11320–033) supplemented with 10% PS at 37 °C for 1 h. The homogenate was filtered with a 70 μm cell strainer and neutralized with F12 medium supplemented with 15% fetal bovine serum (FBS) (Gibco, Carlsbad, CA, USA, #16000–044). After centrifuging at 1100 rpm for 5 min, an ACK lysing buffer (Gibco, Carlsbad, CA, USA, #A10492–01) was added to the supernatant and incubated on ice for 5 min. The supernatant was discarded after centrifuging at 1100 rpm for 5 min once more. The cell pellet was resuspended in F12 medium supplemented with 15% FBS, 1% Penicillin–streptomycin–glutamine (PSG) (Gibco, Carlsbad, CA, USA, #10378–016) and basic fibroblast growth factor (bFGF) (Gibco, Carlsbad, CA, USA, #13256–029, 10 ng/mL). Cells were routinely seeded into a culture plate and cultured in a 37 °C incubator for 1 h. To separate satellite cells, the medium (suspension cells) was collected and transferred to another culture plate coated with 0.1% gelatin.

### 2.3. Treatment with Inhibitor (XAV939) or Activator (CHIR99021)

To regulate the Wnt/β-catenin signaling pathway in PMSCs, XAV939 (Tankyrase inhibitor, Sigma-Aldrich, St Louis, MO, USA, #3004) and CHIR99021 (GSK3β inhibitor, Stemgent, Lexington, MA, USA, #04-0004-02) were used for treatment as an inhibitor and an activator of Wnt/β-catenin signaling pathway, respectively. The control group (SC group) was maintained with growth medium (DMEM/F12 containing 15% FBS, 1% PSG, and 10 ng/mL bFGF), and treatment groups were treated with XAV939 or CHIR99021 at concentrations of 0.5 μM, 1 μM, 3 μM, 5 μM, and 10 μM, respectively. The final treatment dose was selected by comparing the cell viability.

### 2.4. Cell Viability Assay

Cell viabilities were detected with Cell Counting Kit-8 (CCK-8, Dojindo, Kumamoto, Japan, #CK04-11) at 48 and 72 h. Briefly, cells were seeded into 96-well plates at a density of 5 × 10^4^ cells per well and cultured for 48 and 72 h. Cells were then treated with CCK-8 solution following the manufacturer’s instructions, and incubated at 37 °C for 4 h. OD value of each well was measured using a microplate reader at a wavelength of 450 nm.

### 2.5. Cell Proliferation Analysis

PMSCs were seeded into 10 cm^2^ dishes at a density of 1 × 10^6^ cells per dish and cultured for 3 days. Cells were detached with 0.25% trypsin-EDTA and counted under an inverted microscope using a hematocytometer. The experiment was performed in triplicate.

### 2.6. Protein Extraction and Western Blotting

Cells were cultured into 10 cm^2^ dishes at a density of 1 × 10^6^ cells per dish and collected for western blot analysis. Total protein was extracted from satellite cells with RIPA buffer (Biosesang, Sungnam, Republic of Korea) added with protease inhibitor (#A32953, Thermo Fisher, San Jose, CA, USA). Protein concentration was measured using a DC Protein Assay Kit (Bio-Rad, Hercules, CA, USA). Proteins extracted from cells were separated by SDS-PAGE using 12% acrylamide gels and transferred to PVDF (polyvinylidene fluoride) membranes. After blocking with 5% skim milk with TBST buffer at room temperature, membranes were incubated with primary antibodies overnight at 4 °C and then incubated with secondary antibodies at room temperature for 1.5 h. Primary antibodies used in this study were: GAPDH (1:5000, Monoclonal, MA5-15738, Invitrogen, Carlsbad, CA, USA), Pax7 (1:1000, monoclonal, DSHB, Iowa, IA, USA), MyoD (1:1000, polyclonal, 18943-1-AP, Proteintech, Rosemont, IL, USA), Bax (1:1000, monoclonal, sc-7480, Santa Cruz, Dallas, TX, USA), Bcl-2 (1:1000, monoclonal, sc-7382, Santa Cruz, Dallas, TX, USA), β-Catenin (1:1000, monoclonal, #8480T, Cell Signaling Technology, Danvers, MA, USA) and Non-phospho-β-Catenin (1:1000, monoclonal, #8814S, Cell Signaling Technology, Danvers, MA, USA). Secondary antibodies used in this study were goat anti-mouse IgG (HRP-conjugate, #31430, Thermo Fisher, San Jose, CA, USA) and goat anti-rabbit IgG (HRP-conjugate, #31460, Thermo Fisher, San Jose, CA, USA). Protein bands were visualized using an ECL Kit (SuperSignal WestPico Plus, Thermo Fisher, San Jose, CA, USA). Their densities were measured with an iBright CL100 Imaging System (Thermo Fisher, San Jose, CA, USA).

### 2.7. Immunofluorescence Staining of PMSCs

PMSCs were grown on confocal dishes and fixed with 4% paraformaldehyde for 20 min at room temperature. After washing with PBS, cells were permeabilized and blocked with 0.3% Triton X-100 and 3% BSA in PBS for 1 h at room temperature. Cells were then incubated with primary antibodies against anti-Ki67 (Monoclonal, 1:100, GeneTex, Irvine, CA, USA), anti-MyoD (Polyclonal, 1:200, Proteintech, Rosemont, IL, USA), and anti-Pax7 (Monoclonal, 1:50, DSHB, Iowa, IA, USA) at 4 °C overnight. After washing with PBS, primary antibodies were visualized with fluorescently labeled secondary antibodies (Alexa Fluor 488 or 568; Molecular Probes, Eugene, OR, USA). Cells were then stained with 4’-6-diamidino-2-phenylindole (DAPI) for 5 min at room temperature. Fluorescence images were collected using a super-resolution confocal laser scanning microscope (SR-CLSM) installed in the Center for University-Wide Research Facilities (CURF) at Jeonbuk National University.

### 2.8. Flow Cytometry Analysis

Cell cycle and apoptosis analysis were performed with a FACS caliber flow cytometry (Becton, Dickinson Company, Novato, CA, USA) and BD CellQuest Pro software. For cell cycle analysis, samples were fixed with cold 70% ethanol for 5 min at 4 °C. Cells were then stained with diluted propidium iodide (PI) solution (Bio Legend, #421301, San Diego, CA, USA) containing 100 μg/mL of RNase A (Sigma-Aldrich, St Louis, MO, USA, #70856) and immediately analyzed by flow cytometry. Cell apoptosis was detected with a Fluorescein isothiocyanate (FITC) Annexin V Apoptosis Detection Kit and PI (Bio Legend, #640914, San Diego, CA, USA) according to the manufacturer’s instructions. Briefly, cells were detached with 0.25% trypsin-EDTA and washed with 1% BSA in cold PBS. Cells were then double stained with FITC Annexin V and PI solution for 15 min at room temperature in the dark. Stained cells were mixed with Annexin V Binding Buffer and analyzed by flow cytometry.

### 2.9. Gene Expression Analysis by qRT-PCR

Total RNA was extracted from each group using an RNeasy Mini Kit (QIAGEN, Hilden, Germany) following the manufacturer’s protocol. One microgram of extracted RNA was used for reverse transcription with a cDNA synthesis kit (Bioneer, Daejeon, Korea). The expression levels of target genes were analyzed using AMPIGENE^®^ qPCR Green Mix (Enzo, San Diego, CA, USA) on a CFX96™ real-time PCR detection system (Bio-Rad, Hercules, CA, USA). Samples were denatured at 95 °C for 5 min, followed by 40 cycles at 95 °C for 5 s for denaturation and 60 °C for 5 s for annealing/extension. Relative gene expression was calculated as fold change using the 2^−ΔΔCt^ method [19]. The primer sequences of targeted genes used for this study are presented in Table 1.

### 2.10. Library Preparation and Sequencing

Total RNA was extracted from porcine satellite cells using an RNeasy Mini Kit (QI-AGEN, Hilden, Germany). A total of 1 µg RNA was handled for formulating mRNA sequencing library using an MGIEasy RNA Directional Library Prep Kit (MGI, Shenzhen, China) following the manufacturer’s protocol. Very early, purified the poly-A-containing mRNA using magnetic beads obtaining poly-T oligo. During purification, the temperature was raised and the mRNA was split into small sizes. The first and second strand cDNA was synthesized by mRNA transcription. Then, add a single “A” base for ligation with the adaptor. Thus, we made the cDNA library. The library of second-stranded cDNA was measured by QuantiFluor ONE dsDNA System (Promega, Madison, WI, USA). This library was kept in at 37 °Cfor 30 min and then digested at 37 °C for 30 min. In this stage, the library was again incubated with DNB enzyme at 30 °C for 25 min. Finally, the library was measured using QuantiFluor ssDNA system (Promega, Madison, WI, USA).

### 2.11. Preprocessing and Genome Mapping

The existing sequencing connectors and primary quality bases in primary reads were cut with Skewer ver 0.2.2 [20]. The high-quality reads after pruning poor-quality bases and sequencing connectors were plotted to the UCSC susScr11 pig reference genome (https://genome.ucsc.edu/; accessed on 10 November 2020) by a STAR ver 2.5 software [21]. The map was prepared using libraries sequencing with Illumina’s strand-specific library preparation kit.

### 2.12. Quantifying Gene Expression and Differentially Expressed Gene Analysis

In gene expression quantification, Cuffquant of Cufflinks ver 2.2.1 [22] was used with --library-type=fr-firststrand specified. The expressed value was measured in Fragments Per Kilobase of transcript per Million fragments mapped (FPKM) unit. The gene expression of three different groups was analyzed using Cuffdiff in the Cufflinks package [23] with different library options. The gene expression was compared, normalized, and expressed gene was clustered using R. The correlation and hierarchical clustering analysis were performed and visualized in R studio. MDS plots were drawn with R package (ggplot2 package). The scatter plots and *p*-values between two selected values were analyzed using R studio.

### 2.13. Gene Ontology and KEGG Pathway Analysis

The biological functions of differential gene expression in biological conditions, gene set overlapping tests, such as Gene Ontology, KEGG pathways, and other functional gene sets were performed using g: Profiler ver 0.6.7 [24]. To compare the expression of genes involved in the Wnt pathway, FPKM value of each gene was averaged within groups, the fold change of averaged values between two groups was calculated, converted to log_2_ value and was used as the input for pathview (v1.36.1) visualization [25].

### 2.14. Statistical Analysis

Data were analyzed using SAS software version 9.4 (SAS Institute Inc., Cary, NC, USA). Statistical differences were determined with Student’s *t*-test or analysis of variance (ANOVA) followed by Duncan’s Multiple Range Test for post-hoc comparisons. All values are presented as mean ± standard error (SE). All experiments were performed in triplicate.

## 3. Results

### 3.1. Effects of XAV939 and CHIR99021 on Viability and Proliferation of PMSCs

**Figure 1:**
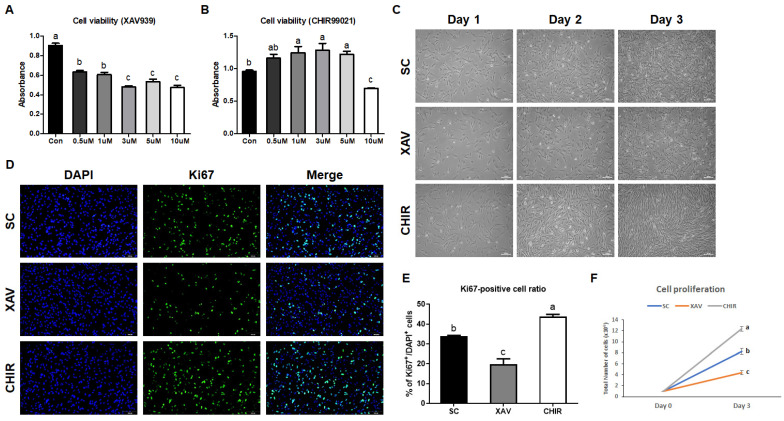
Effect of small molecule addition on porcine muscle satellite cells (PMSCs). (**A**,**B**) Viability of PMSCs according to XAV939 or CHIR99021 doses at 72 h. (**C**) Morphology of PMSCs in each group. (**D**) Immunostaining images of PMSCs for comparison of proliferation rates. Cell nuclei were stained with DAPI (blue) and Ki67 (green). (**E**) The ratio of Ki67^+^/DAPI^+^ cells shown in the immunostaining images. (**F**) Comparison of proliferation rate of PMSCs. SC, control group; XAV, treated with 1 μM of XAV939; CHIR, treated with 3 μM of CHIR99021. Values are presented as mean ± SE. ^a–c^ Different letters represent statistically significant differences among treatment groups (*p* < 0.01). Scale bar, 100 μm.

To evaluate the effects of XAV939 and CHIR99021 on PMSCs, we compared cell viability and proliferation. At 48 (Figure S1) and 72 h, the addition of XAV939 significantly decreased the viability of PMSCs (*p* < 0.01) but the addition of CHIR99021 increased the viability of PMSCs up to the 5 μM dose and remarkably decreased the viability of PMSCs at doses higher than that (*p* < 0.01) (Figure 1A,B). The expression of Ki67, a marker of cell proliferation activity, was significantly increased in the CHIR group (*p* < 0.01) but decreased in the XAV group (*p* < 0.01) compared to the SC (control) group (Figure 1D,E). The result of comparing the morphology of cells (Figure 1C), and proliferation rate by counting the total number of cells was also consistent with the result of Ki67 immunostaining (Figure 1E).

### 3.2. Changes of Pax7 and MyoD Protein and Gene Expression by XAV939 and CHIR99021 in PMSCs

**Figure 2:**
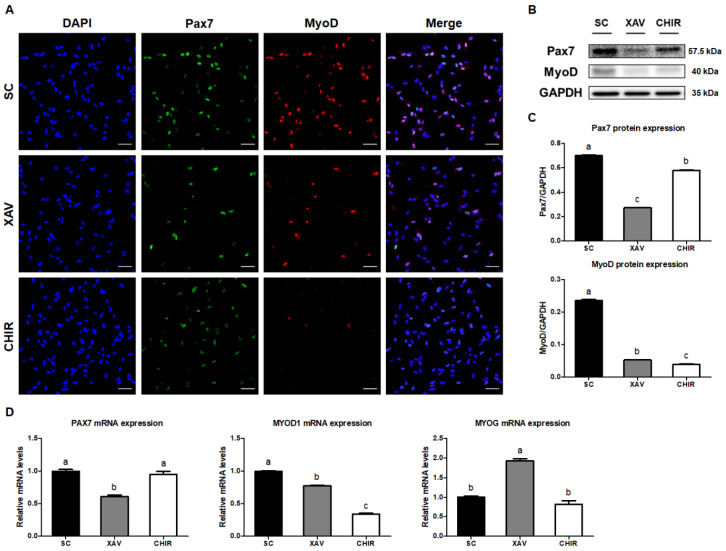
Expression of transcription factors Pax7 and MyoD in PMSCs by addition of Wnt signaling pathway regulators. (**A**) Immunostaining images of PMSCs. Cell nuclei were stained with DAPI (blue), Pax7 (green), and MyoD (red). (**B**) Pax7 and MyoD protein levels in PMSCs. (**C**) Quantitation of Pax7 and MyoD protein levels relative to GAPDH. (**D**) Gene expression levels of *PAX7*, *MYOD1*, and *MYOG* in PMSCs. Values are presented as mean ± SE. ^a–c^ Different letters represent statistically significant differences among treatment groups (*p* < 0.01). Scale bar, 50 μm. Protein marker information used for target protein detection is presented in Supplementary Figure S2. Full-length blots are presented in Supplementary Figure S3.

To investigate the expression of important transcription factors in muscles affected by Wnt signaling pathway regulators, Pax7 and MyoD protein expression levels were analyzed using immunofluorescence staining and Western blot. As a result of immunostaining, Pax7 and MyoD protein levels in PMSCs treated with XAV were decreased compared to those in the SC group, although the number of cells expressing Pax7 and MyoD was similar (Figure 2A). On the other hand, the Pax7 protein level in the CHIR group was decreased compared to that in the SC group, although it was still expressed in many cells, while little MyoD protein was expressed (Figure 2A). The results of Western blot analysis for Pax7 and MyoD protein levels in PMSCs were consistent with the immunostaining results (Figure 2B,C). XAV939 notably reduced both Pax7 and MyoD protein levels compared to the control (SC group) (*p* < 0.01), and CHIR99021 remarkably reduced MyoD expression level while maintaining the expression of Pax7. The gene expression levels of myogenic transcription factors were determined by qRT-PCR. The expression levels of the *PAX7* gene were significantly down-regulated (*p* < 0.01) in PMSCs treated with XAV compared to other groups. The expression levels of the *MYOD1* gene were significantly decreased (*p* < 0.01) in the XAV and CHIR groups, similar to the protein expression results, and the CHIR group showed the lowest gene expression level. In addition, XAV939 significantly increased the expression level of the *MYOG* gene, a myogenic differentiation marker, in PMSCs.

### 3.3. Verification of Effects of XAV939 and CHIR99021 on Cell Cycle and Apoptosis

**Figure 3:**
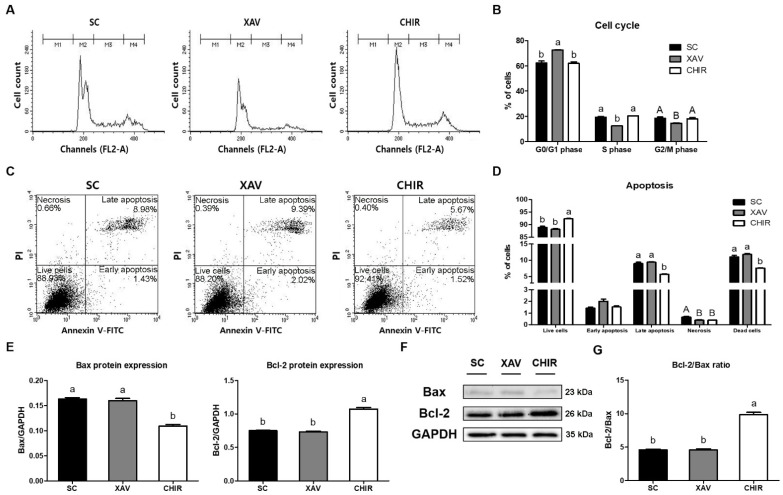
Comparison of cell cycle analysis and apoptosis detection of PMSCs by addition of Wnt signaling pathway regulators. (**A**) Schematic diagram of cell cycle analysis by flow cytometry. (**B**) Cell cycle assay results. (**C**) Dot plot of apoptotic cell death analysis by flow cytometry. (**D**) Apoptosis analysis results. (**E**) Quantitation of Bax and Bcl-2 protein levels relative to GAPDH. (**F**) Protein levels of Bax and Bcl-2 in PMSCs. (**G**) The ratio of Bcl-2/Bax. Dead cells = cells during early apoptosis + late apoptosis + necrosis. Values are presented as mean ± SE. ^a,b^ Different letters represent statistically significant differences among treatment groups (*p* < 0.01). ^A,B^ Different letters represent statistically significant differences among treatment groups (*p* < 0.05). Full-length blots are presented in Supplementary Figure S4.

The effects of the Wnt signaling pathway inhibitor and activator on the cell cycle and apoptosis of PMSCs were determined using a flow cytometer. The percentage of PMSCs in the G0/G1 phase in the XAV group was significantly higher than that in the SC and CHIR groups (*p* < 0.01) (Figure 3A,B). On the other hand, percentages of PMSCs in the S phase (*p* < 0.01) and G2/M phase (*p* < 0.05) in the SC and CHIR groups were significantly higher than those in the XAV group (Figure 3A,B). The addition of XAV939 or CHIR99021 also affected the apoptosis of PMSCs. The percentage of live cells was the highest in the CHIR group (*p* < 0.01). Proportions of late apoptosis and total dead cells were significantly higher in the SC and XAV groups than that in the CHIR group (*p* < 0.01) (Figure 3C,D). The percentage of cell necrosis was significantly higher in the SC group than in the other groups (*p* < 0.05). However, there was no significant difference in the percentage of early apoptosis (Figure 3C,D). The expression level of Bax, a pro-apoptotic protein, was significantly higher in the SC and XAV groups than that in the CHIR group. The expression level of Bcl-2, an anti-apoptotic protein, was significantly higher in the CHIR group than in the other groups (*p* < 0.01) (Figure 3E–G).

### 3.4. Comparison of Gene Expression Patterns in PMSCs

**Figure 4:**
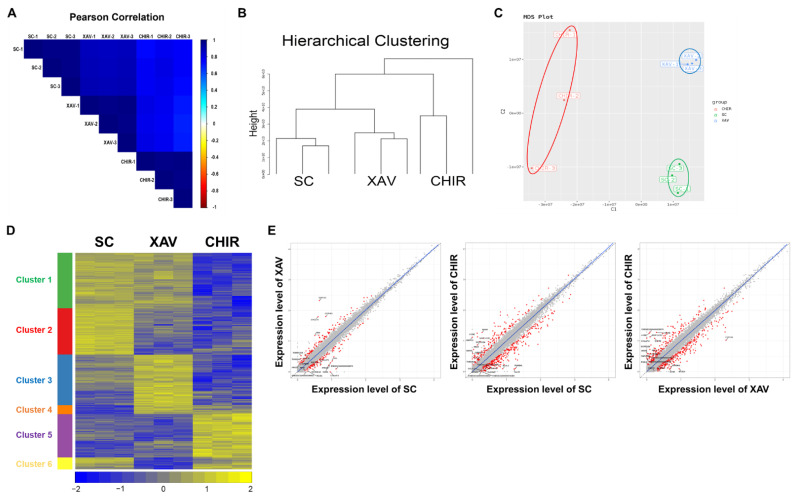
Gene expression pattern of PMSCs. (**A**) Pearson correlation analysis of SC, XAV, and CHIR groups. (**B**) Hierarchical clustering analysis of SC, XAV, and CHIR groups. (**C**) Multidimensional scaling (MDS) plot of SC, XAV, and CHIR groups. (**D**) Heat map of gene expression patterns in SC, XAV, and CHIR groups. (**E**) Scatter plots comparing SC and XAV, SC and CHIR, and XAV and CHIR groups. Related terms for each cluster in the heat map analysis result are presented in Supplementary Figure S5.

To compare gene expression patterns in PMSCs after XAV939 or CHIR99021 treatment, we obtained gene expression profiles through RNA sequencing analysis (Figure 4A–E). Based on gene expression profiles and Pearson correlation analysis data, cells were clustered (Figure 4A). Hierarchical clustering and multidimensional scaling (MDS) plot data showed that the gene expression patterns in each group were distinct from each other (Figure 4B,C). As a result of heat map analysis, gene expression patterns were divided into six clusters, among which clusters showing opposite expression patterns (clusters 1, 3, 5, and 6) by XAV939 or CHIR99021 treatment were very interesting (Figure 4D). Genes in cluster 1 were significantly enriched in terms of “muscle organ development”, “negative regulation of cell proliferation”, “skeletal muscle cell differentiation”, “skeletal muscle fiber development”, and “sarcomere organization” (Figure S5). Moreover, genes in cluster 3 were identified as being related to “positive regulation of protein phosphorylation”, genes in cluster 5 to “negative regulation of canonical Wnt signaling pathway”, and genes in cluster 6 to “positive regulation of canonical Wnt signaling pathway” (Figure S5). Scatter plot analysis results also demonstrated that each group had different gene expression patterns (Figure 4E).

### 3.5. Identification of Differentially Expressed Genes in XAV939 and CHIR99021 Treated PMSCs

**Figure 5:**
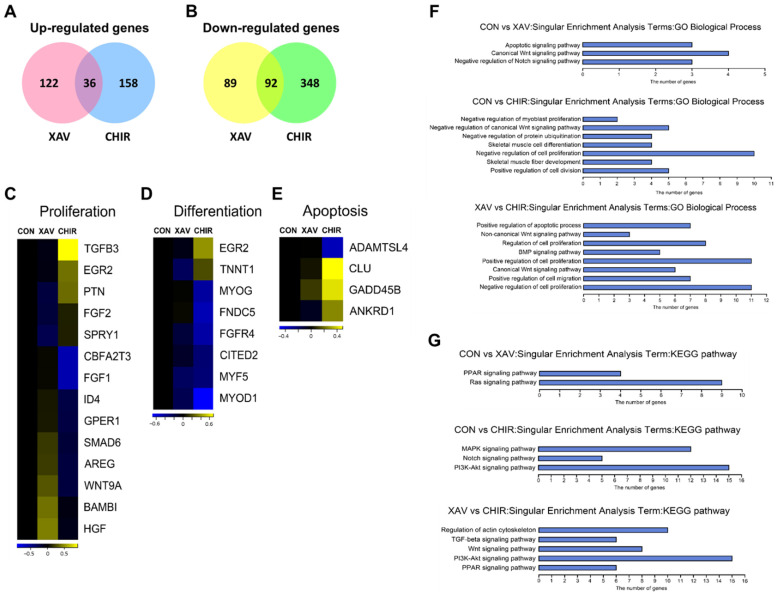
Differentially expressed genes (DEGs) in XAV939 and CHIR99021 treated PMSCs. (**A**) Up-regulated genes in XAV or CHIR groups. (**B**) Down-regulated genes in XAV or CHIR groups. (**C**) Heat map of muscle cell proliferation-related genes in PMSCs. (**D**) Heat map of muscle cell differentiation-related genes in PMSCs. (**E**) Heat map of apoptosis-related genes in PMSCs. (**F**) Gene ontology (GO) term and (**G**) KEGG pathway analysis of DEGs selected from PMSCs. d CHIR groups.

To identify differentially expressed genes (DEGs) in PMSCs using criteria of |Fold-Change| ≥ 2 and *p*-value ≤ 0.05, we compared expression levels of genes between XAV939- and CHIR99021-treated cells and the SC group of cells. Among the selected DEGs, 158 and 194 genes were up-regulated in the XAV and CHIR groups, respectively, compared to the SC group (Figure 5A). In addition, 181 and 440 genes were down-regulated in the XAV group and the CHIR group, respectively, compared to the SC group (Figure 5B). Expression patterns of proliferation-related genes were different among the three groups (Figure 5C). Of these genes, *SMAD6*, *AREG*, *WNT9A*, *BAMBI*, and *HGF* genes were up-regulated in the XAV group and *TGFB3*, *EGR2*, and *PTN* genes were up-regulated in the CHIR group compared to those in the other two groups. In contrast, expression levels of the *CBFA2T3* and *FGF1* genes were lower in the CHIR group than that in the SC and XAV groups. Among differentiation-related genes, *EGR2* and *TNNT1* genes were highly expressed in the CHIR group. However, most genes, including representative muscle differentiation markers such as *MYOG* and *MYOD1*, were expressed lower in the CHIR group than in the other two groups (Figure 5D). Expression patterns of apoptosis-related genes in the XAV group were similar to those in the SC group (Figure 5E). By contrast, the *ADAMTSL4* gene was down-regulated while *CLU*, *GADD45B*, and *ANKRD1* genes were up-regulated in the CHIR group.

To classify functions of DEGs identified in the XAV and CHIR groups, we performed Gene Ontology (GO) term and KEGG pathway analysis using DAVID bioinformatics resources (https://david.ncifcrf.gov/; accessed on 29 September 2022). The total numbers of DEGs for comparisons of SC vs. XAV, SC vs. CHIR, and XAV vs. CHIR were 339, 634, and 633, respectively (|Fold-Change| ≥ 2 and *p*-value ≤ 0.05). DEGs in comparison to SC vs. XAV were significantly enriched for GO terms of “apoptotic signaling pathway”, “canonical Wnt signaling pathway”, and “negative regulation of Notch signaling pathway” (Figure 5F). The most significant enriched GO terms in comparison of SC vs. CHIR were “negative regulation of myoblast proliferation”, “negative regulation of canonical Wnt signaling pathway”, “negative regulation of protein ubiquitination”, “skeletal muscle cell differentiation”, “negative regulation of cell proliferation”, “skeletal muscle fiber development”, and “positive regulation of cell division” (Figure 5F). DEGs between the XAV and CHIR groups were significantly enriched for terms of “positive regulation of apoptotic process”, “non-canonical Wnt signaling pathway”, “regulation of cell proliferation”, “BMP signaling pathway”, “positive regulation of cell proliferation”, “canonical Wnt signaling pathway”, “positive regulation of cell migration”, and “negative regulation of cell proliferation” (Figure 5F). Our results in the GO analysis showed that DEGs in XAV939- and CHIR99021-treated PMSCs were mainly involved in muscle cell development (proliferation and differentiation), apoptosis, and the Wnt signaling pathway. KEGG pathway analysis revealed that DEGs for comparison of SC vs. XAV were significantly enriched for the “PPAR and Ras signaling pathway”, whereas DEGs for comparison of SC vs. CHIR included “MAPK, Notch, and PI3K-Akt signaling pathway” (Figure 5G). In addition, DEGs between the XAV and CHIR groups were significantly enriched for “regulation of actin cytoskeleton” and “TGF-beta, Wnt, PI3K-Akt, and PPAR signaling pathway” (Figure 5G).

### 3.6. Validation of Changes in Wnt Signaling-Related Factors in XAV939- and CHIR99021-Treated PMSCs

**Figure 6:**
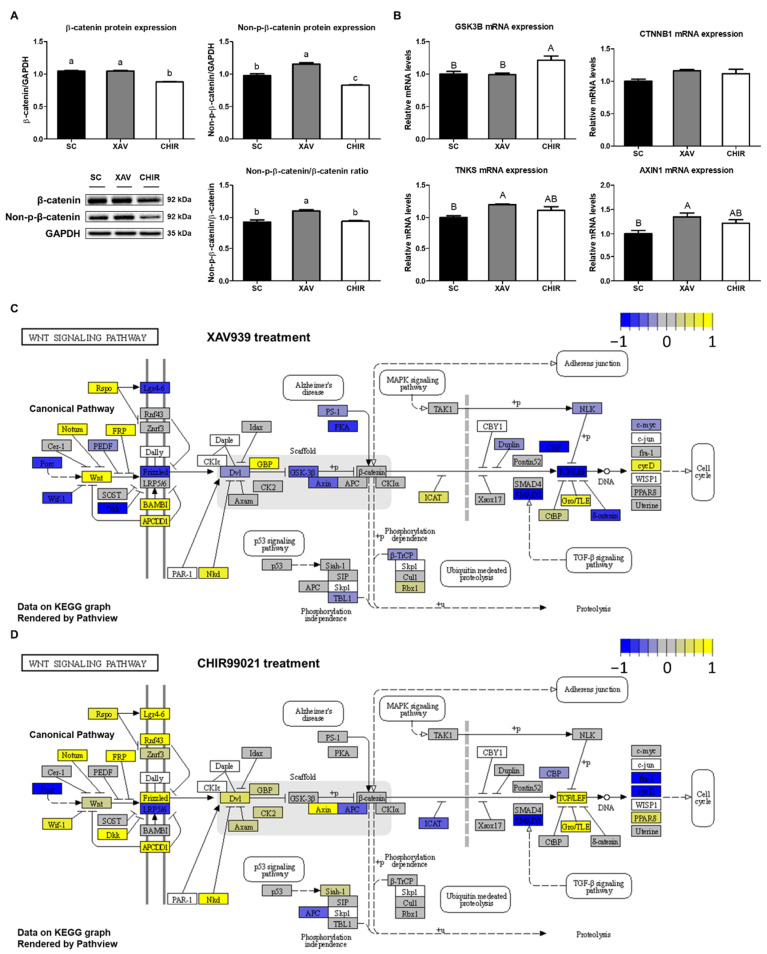
Changes in target factors in PMSCs by addition of Wnt signaling pathway regulators. (**A**) β-Catenin and non-phospho-β-Catenin (active β-Catenin) protein levels and non-phospho-β-Catenin/β-Catenin ratio in PMSCs. (**B**) Quantitative gene expression levels of *GSK3B*, *CTNNB1*, *TNKS*, and *AXIN1* in PMSCs. (**C**) Changes in expression of Wnt signaling-related factors in XAV939 treated cells. (**D**) Changes in expression of Wnt signaling-related factors in CHIR99021 treated cells. Values are presented as mean ± SE. ^a–c^ Different letters represent statistically significant differences among treatment groups (*p* < 0.01). ^A,B^ Different letters represent statistically significant differences among treatment groups (*p* < 0.05). Full-length blots are presented in Supplementary Figure S6.

We verified that treatment with XAV939 and CHIR99021 altered the expression of Wnt signaling-related factors in PMSCs. The expression level of the β-catenin protein was significantly decreased (*p* < 0.01) in the CHIR group (Figure 6A), and the expression level of the non-phospho-β-catenin protein increased (*p* < 0.01) in the XAV group and decreased (*p* < 0.01) in the CHIR group (Figure 6A). The ratio of non-phospho-β-catenin/β-catenin was significantly higher (*p* < 0.01) in the XAV group than that in the other groups (Figure 6A). As a result of gene expression analysis for target factors of Wnt signal regulators, the expression level of the *GSK3B* gene was significantly increased (*p* < 0.05) in the CHIR group and there was no significant difference in the expression level of the *CTNNB1* gene (Figure 6B). The expression levels of the *TNKS* and *AXIN1* genes were the highest (*p* < 0.05) in the XAV group (Figure 6B). In addition, we compared the expression of genes involved in the canonical Wnt signaling pathway (Figure 6C,D). In XAV939-treated PMSCs, the expression of canonical signaling pathway-related factors, such as Wif-1, Dkk, Frizzled, and Dvl, was down-regulated. In addition, XAV939 reduced the expression of the transcription factor TCF/LEF, which is downstream of the signaling pathway (Figure 6C). However, in CHIR99021-treated PMSCs, the expression of upstream factors of the Wnt signaling pathway was up-regulated, and as a result, the expression of TCF/LEF was increased (Figure 6D).

## 4. Discussion

Canonical Wnt signaling is an important regulator of skeletal muscle satellite cell development and regeneration [15,26]. We found that the proliferation of PMSCs was controlled by small molecules known to regulate the Wnt signaling pathway. XAV939 is an inhibitor of the Wnt signaling pathway that inhibits tankyrase (TNKS) [27], which acts as an accelerant of the Wnt signal by mediating poly-ADP-ribosylation (PARylation) of Axin, a cytosolic scaffold of the β-catenin destruction complex. CHIR99021 activates the Wnt signaling pathway by inhibiting GSK3β, which induces degradation by phosphorylating β-catenin. In this study, we confirmed that XAV939 or CHIR99021 treatment inhibited or promoted the activity of the Wnt signaling pathway by regulating the expression of TCF/LEF, a key transcription factor, through expression comparison of Wnt signaling-related genes and KEGG pathway analysis. In pig intestinal epithelial cells, recombinant human (rh) Wnt3a supplementation activated Wnt/β-catenin signaling and increased cell proliferation, whereas XAV939 supplementation inhibited Wnt/β-catenin signaling and cell proliferation [28]. XAV939 also attenuated the proliferation and migration of murine vascular smooth muscle cells (VSMCs) and promoted cell cycle arrest in the G0/G1 phase of VSMCs [29]. However, inhibition of GSK3β by CHIR99021 can promote the self-renewal of mouse embryonic stem cells (ESCs) by regulating multiple signaling pathways [30] and it can increase the proliferation of neural stem cells [31]. It was also reported that CHIR99021 can promote the proliferation of cardiomyocytes in humans [32] and improve cell proliferation while maintaining the undifferentiated state of human induced pluripotent stem cells (hiPSCs) [33]. The effect of Wnt signaling on the stemness of stem cells appears to be different depending on the type of stem cells. In the present study, we found that the addition of XAV939 or CHIR99021 to PMSCs decreased or increased the proliferation rate, respectively, compared to the control (SC group). In addition, in cells treated with XAV939, expression levels of Pax7 and MyoD were remarkably decreased. However, cells treated with CHIR99021 showed little expression of MyoD while maintaining the expression of Pax7. Transcription factor Pax7 is essential for the maintenance and self-renewal of satellite cells. It can induce the proliferation of myoblasts and delay their differentiation [34,35]. Typically, activated satellite cells co-express Pax7 and MyoD, while quiescent satellite cells express Pax7 only [36]. The expression of stabilized β-catenin by inhibition of GSK3β in activated satellite cells can down-regulate the expression of MyoD and myogenin, and β-catenin can advance the self-renewal of satellite cells [37]. These results indicate that CHIR99021 might induce stabilization of β-catenin in the cytoplasm by inhibiting GSK3β, thereby improving the self-renewal and proliferation capacity of muscle satellite cells and maintaining the pool of satellite cells. Maintaining the stemness of satellite cells in vitro is helpful for the mass proliferation of satellite cells, which can be a key technology for cultured meat production.

We also found that CHIR99021 could increase the viability and suppress apoptosis of PMSCs. It was reported that CHIR99021 can activate the Wnt signaling pathway and inhibit apoptosis of porcine-induced pluripotent stem cells (piPSCs) [38]. Combined use of CHIR99021 and XAV939 can improve the viability of porcine pluripotent stem cell lines (pPSCs) in a PI3K/Akt pathway-dependent way [39]. It was reported that β-catenin also can induce the expression of Bcl-2 through a downstream pathway [40]. Moreover, myoblasts lacking the *MYOD1* gene in mice have superior self-renewal efficiency than wild-type myoblasts [41]. Of these myoblasts, many anti-apoptosis genes are up-regulated, whereas apoptosis-inducing genes are down-regulated, meaning that MyoD^-/-^ myoblasts have higher resistance to programmed cell death and cell viability than other types of myoblasts [41]. In the present study, CHIR99021 not only reduced the expression of the MyoD protein and gene but also suppressed apoptosis by regulating the expression of apoptotic markers (such as Bax and Bcl-2) and apoptosis-related genes.

A previous study has reported that stabilization of β-catenin by CHIR99021 in mouse ESC affects not only the Wnt/β-catenin pathway but also the TGF-β, Notch, and MAPK pathways [42]. Wnt/β-catenin signaling can increase the expression of Notch signaling [43]. Notch signaling is essential for the development, proliferation, and apoptosis of normal cells [44]. Activated Notch signaling can up-regulate the expression of programmed cell death inhibitory genes, such as Bcl-2 and Bcl-XL [45,46]. In addition, Li, Zhang [47] have suggested that CHIR99021 up-regulated the Notch3 protein expression and its downstream genes and accelerated cell proliferation in human lung cancer cells, although the regulation of CHIR99021 on apoptosis might vary depending on the cell type. Notch signaling also can activate the PI3K/Akt pathway through the activation of HES1 [48]. The PI3K/Akt and Ras/MAPK signaling pathways were reported to contribute to the regulation of cell growth, proliferation, differentiation, and survival [13,49,50]. Inhibition of these pathways can reduce cell growth and proliferation and induce apoptosis [51,52]. It was reported that co-treatment with CHIR99021 and cAMP activator forskolin in glioma can cause changes in the Ras/MAPK and PI3K/Akt signaling pathways involved in cell growth and proliferation [53]. In addition, simultaneous activation of the Wnt/β-catenin and PI3K/Akt signaling pathways using CHIR99021 and insulin can induce self-renewal and expansion of hematopoietic stem cells [54]. Here, we found that the addition of XAV939 or CHIR99021 to PMSCs could regulate the Wnt/β-catenin, Ras/MAPK, PI3K/Akt, Notch, and TGF-β signaling pathways. These results suggest that CHIR99021 is involved in the proliferation and survival of PMSCs through various signaling pathways in addition to Wnt/β-catenin signaling.

**Figure 7:**
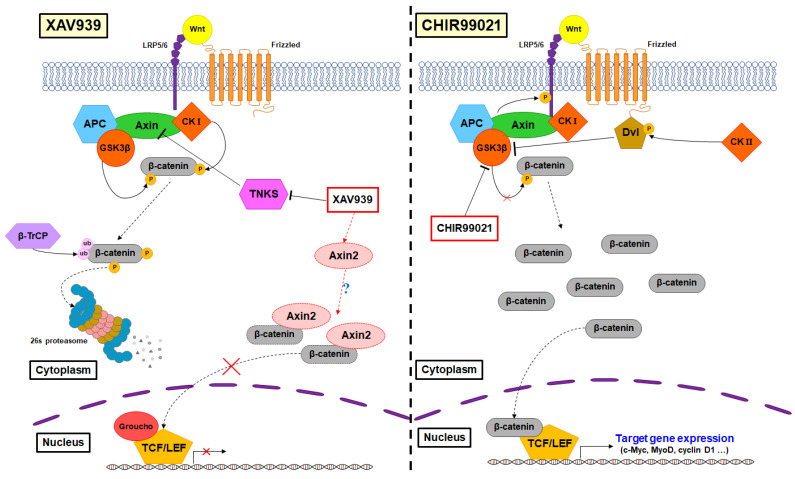
Systematic mechanisms of β-catenin in PMSCs.

Activation of Wnt/β-catenin signaling produces heterogeneous effects depending on cell type and culture conditions, which are still unclear. Some studies have reported that activation of the Wnt/β-catenin signaling pathway promotes the self-renewal of human ESCs [55,56], but other studies have suggested that it induces differentiation of human ESCs [57,58]. Regarding the disparate role of activated Wnt/β-catenin signaling, Kim et al. [59] reported that the self-renewal or differentiation of stem cells was regulated depending on whether stabilized β-catenin was translocated to the nucleus or maintained in the cytoplasm. They found that in mouse EpiSCs and human ESCs, self-renewal was promoted when stabilized β-catenin is retained in the cytoplasm, and differentiation was induced when β-catenin translocates into the nucleus and binds to TCF [59]. In addition, Kim et al. [59] demonstrated that XAV939 stabilizes Axin2, and the stabilized Axin2 binds to β-catenin to help retain it in the cytoplasm. In contrast, we found that GSK3β inhibitor treatment activates Wnt/β-catenin signaling and increases the expression of TCF/LEF, thereby promoting the proliferation and self-renewal of PMSCs. Our findings are consistent with previous studies showing that self-renewal of mouse ESCs is maintained when Wnt signaling is activated and β-catenin binds to TCF [60,61]. These previous studies support our results that inhibition of GSK3β by the addition of CHIR99021 stabilized β-catenin in the cytoplasm and promoted its translocation into the nucleus, increasing TCF/LEF expression (Figure 7). Furthermore, the mechanism by which the addition of XAV939 to PMSCs inhibits Wnt/β-catenin signaling (increased β-catenin expression and decreased TCF/LEF expression) could be explained. Most studies on Wnt signaling were conducted on pluripotent stem cells from humans or mice but this study differs from previous studies in that it used muscle satellite cells, which are adult stem cells of porcine, an animal that can be used for meat. However, further studies are needed on how CHIR99021 and XAV939 affect various signaling pathways in PMSCs.

## 5. Conclusions

We showed that CHIR99021, a GSK3β inhibitor, could promote the proliferation of PMSCs and contribute to the maintenance of satellite cells by inhibiting MyoD expression while maintaining the expression of Pax7. In addition, we showed that CHIR99021 might suppress the apoptosis of PMSCs by regulating the expression of apoptosis-related proteins and genes. We also identified gene expression patterns and signaling pathways related to the maintenance and survival of satellite cells through RNA sequencing analysis. Our findings can be used for the mass proliferation of muscle stem cells for the production of cultured meat and can contribute to physiological and medical research by simulating the process of muscle regeneration and development in vitro.

## Figures and Tables

**Table 1:** The primer sequences for qRT-PCR.

Gene Name	Primer Sequences	Accession Number	Length (bp)
*GAPDH*	F: 5′-ACCCAGAAGACTGTGGATGG-3′	NM_001206359.1	79
	R: 5′-AAGCAGGGATGATGTTCTGG-3′		
*PAX7*	F: 5′-TCCAGCTACTCCGACAGCTT-3′	XM_021095458.1	100
	R: 5′-TGCTCAGAATGCTCATCACC-3′		
*MYOD1*	F: 5′-GTGCAAACGCAAGACCACTA-3′	NM_001002824.1	128
	R: 5′-GCTGATTCGGGTTGCTAGAC-3′		
*MYOG*	F: 5′-CCACTTCTATGACGGGGAAA-3′	NM_001012406.1	203
	R: 5′-GGTCCACAGACACGGACTTC-3′		
*GSK3B*	F: 5′-TCCTAGGGACACCAACAAGG-3′	NM_001128443.1	190
	R: 5′-CAAGCTTCCAGTGGTGTCAA-3′		
*CTNNB1*	F: 5′-CATCATGCGTTCTCCTCAGA-3′	NM_214367.1	188
	R: 5′-AATCCACTGGTGAACCAAGC-3′		
*TNKS*	F: 5′-AGAGGCCTTACCCACCTGTT-3′	XM_003133396.5	144
	R: 5′-CCTCCTACTGCCAGTTCTGC-3′		
*AXIN1*	F: 5′-CTTCTGCTCTGGGAAAGGTG-3′	XM_021086926.1	171
	R: 5′-TTTATCCCGTCCTGATCGTC-3′		

## Data Availability

Not applicable.

## Supplementary Materials

The following supporting information can be downloaded at: https://www.mdpi.com/article/10.3390/ani12233328/s1, Figure S1: Viability of porcine muscle satellite cells (PMSCs) according to XAV939 or CHIR99021 dose at 48 h. Values are presented as mean ± SE. a-c Different letters represent statistically significant differences among treatment groups (*p* < 0.01); Figure S2: Protein marker information (molecular weight) used for Western blot analysis. Proteins extracted from cells were separated by SDS-PAGE using 12% acrylamide gels, and each protein expression was normalized by GAPDH. Product; Smart Color Protein Marker (Pre-stained), ELPISBIO, EBM-2000; Figure S3: Western blotting band of Pax7, MyoD, and GAPDH in PMSCs. The cropped gel image used in the main text is highlighted in blue; Figure S4: Western blotting band of Bax, Bcl-2, and GAPDH in PMSCs. The cropped gel image used in the main text is highlighted in blue; Figure S5: Related terms for each cluster in the heat map analysis.; Figure S6: Western blotting band of β-catenin, non-phospho-β-catenin, and GAPDH in PMSCs. The cropped gel image used in the main text is highlighted in blue.

## References

[1] Post M.J. (2012). Cultured meat from stem cells: Challenges and prospects. Meat Sci..

[2] Post M.J., Levenberg S., Kaplan D.L., Genovese N., Fu J., Bryant C.J., Negowetti N., Verzijden K., Moutsatsou P. (2020). Scientific, sustainability and regulatory challenges of cultured meat. Nat. Food.

[3] Morgan J., Partridge T. (2020). Skeletal muscle in health and disease. Dis. Model. Mech..

[4] Dumont N.A., Wang Y.X., Rudnicki M.A. (2015). Intrinsic and extrinsic mechanisms regulating satellite cell function. Development.

[5] Chal J., Pourquié O. (2017). Making muscle: Skeletal myogenesis in vivo and in vitro. Development.

[6] Gilbert P.M., Havenstrite K.L., Magnusson K.E., Sacco A., Leonardi N.A., Kraft P., Nguyen N.K., Thrun S., Lutolf M.P., Blau H.M. (2010). Substrate elasticity regulates skeletal muscle stem cell self-renewal in culture. Science.

[7] Vitello L., Radu C., Malerba A., Segat D., Cantini M., Carraro U., Baroni M.D. (2004). Enhancing myoblast proliferation by using myogenic factors: A promising approach for improving fiber regeneration in sport medicine and skeletal muscle diseases. Basic Appl. Myol.

[8] Nicola N.A., Babon J.J. (2015). Leukemia inhibitory factor (LIF). Cytokine Growth Factor Rev..

[9] Thornton K., Kamange-Sollo E., White M.E., Dayton W.R. (2015). Role of G protein–coupled receptors (GPCR), matrix metalloproteinases 2 and 9 (MMP2 and MMP9), heparin-binding epidermal growth factor–like growth factor (hbEGF), epidermal growth factor receptor (EGFR), erbB2, and insulin-like growth factor 1 receptor (IGF-1R) in trenbolone acetate–stimulated bovine satellite cell proliferation. J. Anim. Sci..

[10] Pawlikowski B., Vogler T.O., Gadek K., Olwin B.B. (2017). Regulation of skeletal muscle stem cells by fibroblast growth factors. Dev. Dyn..

[11] Ben-Arye T., Levenberg S. (2019). Tissue engineering for clean meat production. Front. Sustain. Food Syst..

[12] Zammit P.S. (2008). All muscle satellite cells are equal, but are some more equal than others?. J. Cell Sci..

[13] Koveitypour Z., Panahi F., Vakilian M., Peymani M., Forootan F.S., Esfahani M.H.N., Ghaedi K. (2019). Signaling pathways involved in colorectal cancer progression. Cell Biosci..

[14] Ten Berge D., Kurek D., Blauwkamp T., Koole W., Maas A., Eroglu E., Siu R.K., Nusse R. (2011). Embryonic stem cells require Wnt proteins to prevent differentiation to epiblast stem cells. Nat. Cell Biol..

[15] Holland J.D., Klaus A., Garratt A.N., Birchmeier W. (2013). Wnt signaling in stem and cancer stem cells. Curr. Opin. Cell Biol..

[16] Fleming H.E., Janzen V., Celso C.L., Guo J., Leahy K.M., Kronenberg H.M., Scadden D.T. (2008). Wnt signaling in the niche enforces hematopoietic stem cell quiescence and is necessary to preserve self-renewal in vivo. Cell Stem Cell.

[17] Kim C.-H., Neiswender H., Baik E.J., Xiong W.C., Mei L. (2008). β-Catenin interacts with MyoD and regulates its transcription activity. Mol. Cell. Biol..

[18] Reya T., Clevers H. (2005). Wnt signalling in stem cells and cancer. Nature.

[19] Livak K.J., Schmittgen T.D. (2001). Analysis of relative gene expression data using real-time quantitative PCR and the 2−ΔΔCT method. Methods.

[20] Jiang H., Lei R., Ding S.-W., Zhu S. (2014). Skewer: A fast and accurate adapter trimmer for next-generation sequencing paired-end reads. BMC Bioinform..

[21] Dobin A., Davis C.A., Schlesinger F., Drenkow J., Zaleski C., Jha S., Batut P., Chaisson M., Gingeras T.R. (2013). STAR: Ultrafast universal RNA-seq aligner. Bioinformatics.

[22] Trapnell C., Williams B.A., Pertea G., Mortazavi A., Kwan G., Van Baren M.J., Salzberg S.L., Wold B.J., Pachter L. (2010). Transcript assembly and quantification by RNA-Seq reveals unannotated transcripts and isoform switching during cell differentiation. Nat. Biotechnol..

[23] Trapnell C., Hendrickson D.G., Sauvageau M., Goff L., Rinn J.L., Pachter L. (2013). Differential analysis of gene regulation at transcript resolution with RNA-seq. Nat. Biotechnol..

[24] Reimand J., Arak T., Adler P., Kolberg L., Reisberg S., Peterson H., Vilo J. (2016). g: Profiler—A web server for functional interpretation of gene lists (2016 update). Nucleic Acids Res..

[25] Luo W., Brouwer C. (2013). Pathview: An R/Bioconductor package for pathway-based data integration and visualization. Bioinformatics.

[26] Murphy M.M., Keefe A.C., Lawson J.A., Flygare S.D., Yandell M., Kardon G. (2014). Transiently active Wnt/β-catenin signaling is not required but must be silenced for stem cell function during muscle regeneration. Stem Cell Rep..

[27] Huang S.-M.A., Mishina Y.M., Liu S., Cheung A., Stegmeier F., Michaud G.A., Charlat O., Wiellette E., Zhang Y., Wiessner S. (2009). Tankyrase inhibition stabilizes axin and antagonizes Wnt signalling. Nature.

[28] Li X.-G., Wang Z., Chen R.-Q., Fu H.-L., Gao C.-Q., Yan H.-C., Xing G.-X., Wang X.-Q. (2018). LGR5 and BMI1 increase pig intestinal epithelial cell proliferation by stimulating WNT/β-catenin signaling. Int. J. Mol. Sci..

[29] Chen L., Zhuang J., Singh S., Wang K., Xiong M., Xu D., Chen W., Pang J., Xu Y., Li X. (2016). XAV939 inhibits intima formation by decreasing vascular smooth muscle cell proliferation and migration through blocking Wnt signaling. J. Cardiovasc. Pharmacol..

[30] Ye S., Tan L., Yang R., Fang B., Qu S., Schulze E.N., Song H., Ying Q., Li P. (2012). Pleiotropy of glycogen synthase kinase-3 inhibition by CHIR99021 promotes self-renewal of embryonic stem cells from refractory mouse strains. PLoS ONE.

[31] Pachenari N., Kiani S., Javan M. (2017). Inhibition of glycogen synthase kinase 3 increased subventricular zone stem cells proliferation. Biomed. Pharmacother..

[32] Wang S., Ye L., Li M., Liu J., Jiang C., Hong H., Zhu H., Sun Y. (2016). GSK-3β inhibitor CHIR-99021 promotes proliferation through upregulating β-catenin in neonatal atrial human cardiomyocytes. J. Cardiovasc. Pharmacol..

[33] Yoda K., Ohnuki Y., Kurosawa H. (2019). Optimization of the treatment conditions with glycogen synthase kinase-3 inhibitor towards enhancing the proliferation of human induced pluripotent stem cells while maintaining an undifferentiated state under feeder-free conditions. J. Biosci. Bioeng..

[34] Le Grand F., Rudnicki M.A. (2007). Skeletal muscle satellite cells and adult myogenesis. Curr. Opin. Cell Biol..

[35] Almeida C.F., Fernandes S.A., Ribeiro Junior A.F., Keith Okamoto O., Vainzof M. (2016). Muscle satellite cells: Exploring the basic biology to rule them. Stem Cells Int..

[36] Zammit P.S., Relaix F., Nagata Y., Ruiz A.P., Collins C.A., Partridge T.A., Beauchamp J.R. (2006). Pax7 and myogenic progression in skeletal muscle satellite cells. J. Cell Sci..

[37] Perez-Ruiz A., Ono Y., Gnocchi V.F., Zammit P.S. (2008). β-catenin promotes self-renewal of skeletal-muscle satellite cells. J. Cell Sci..

[38] Genovese N.J., Domeier T.L., Telugu B.P.V., Roberts R.M. (2017). Enhanced development of skeletal myotubes from porcine induced pluripotent stem cells. Sci. Rep..

[39] Li Y., Wu S., Li X., Guo S., Cai Z., Yin Z., Zhang Y., Liu Z. (2020). Wnt signaling associated small molecules improve the viability of pPSCs in a PI3K/Akt pathway dependent way. J. Cell. Physiol..

[40] Wu W.K., Wang X.J., Cheng A.S., Luo M.X., Ng S.S., To K.F., Chan F.K., Cho C.H., Sung J.J., Yu J. (2013). Dysregulation and crosstalk of cellular signaling pathways in colon carcinogenesis. Crit. Rev. Oncol./Hematol..

[41] Asakura A., Hirai H., Kablar B., Morita S., Ishibashi J., Piras B.A., Christ A.J., Verma M., Vineretsky K.A., Rudnicki M.A. (2007). Increased survival of muscle stem cells lacking the MyoD gene after transplantation into regenerating skeletal muscle. Proc. Natl. Acad. Sci. USA.

[42] Wu Y., Ai Z., Yao K., Cao L., Du J., Shi X., Guo Z., Zhang Y. (2013). CHIR99021 promotes self-renewal of mouse embryonic stem cells by modulation of protein-encoding gene and long intergenic non-coding RNA expression. Exp. Cell Res..

[43] Bertrand F.E., Angus C.W., Partis W.J., Sigounas G. (2012). Developmental pathways in colon cancer: Crosstalk between WNT, BMP, Hedgehog and Notch. Cell Cycle.

[44] Previs R.A., Coleman R.L., Harris A.L., Sood A.K. (2015). Molecular pathways: Translational and therapeutic implications of the Notch signaling pathway in cancer. Clin. Cancer Res..

[45] Ramana K.V., Tammali R., Srivastava S.K. (2010). Inhibition of Aldose Reductase Prevents Growth Factor–Induced G1-S Phase Transition through the AKT/Phosphoinositide 3-Kinase/E2F-1 Pathway in Human Colon Cancer Cells. Mol. Cancer Ther..

[46] Hristova N.R., Tagscherer K.E., Fassl A., Kopitz J., Roth W. (2013). Notch1-dependent regulation of p27 determines cell fate in colorectal cancer. Int. J. Oncol..

[47] Li C., Zhang S., Lu Y., Zhang Y., Wang E., Cui Z. (2013). The roles of Notch3 on the cell proliferation and apoptosis induced by CHIR99021 in NSCLC cell lines: A functional link between Wnt and Notch signaling pathways. PLoS ONE.

[48] Rajendran D.T., Subramaniyan B., Ganeshan M. (2017). Role of Notch signaling in colorectal cancer. Role of Transcription Factors in Gastrointestinal Malignancies.

[49] Liu P., Cheng H., Roberts T.M., Zhao J.J. (2009). Targeting the phosphoinositide 3-kinase pathway in cancer. Nat. Rev. Drug Discov..

[50] Świderska E., Strycharz J., Wróblewski A., Szemraj J., Drzewoski J., Śliwińska A. (2018). Role of PI3K/AKT pathway in insulin-mediated glucose uptake. Blood Glucose Levels.

[51] Temiz T.K., Altun A., Turgut N., BALCI E. (2014). Investigation of the effects of drugs effective on PI3K-AKT signaling pathway in colorectal cancer alone and in combination. Cumhur. Tıp Derg..

[52] Su Y., Li X., Ma J., Zhao J., Liu S., Wang G., Edwards H., Taub J.W., Lin H., Ge Y. (2018). Targeting PI3K, mTOR, ERK, and Bcl-2 signaling network shows superior antileukemic activity against AML ex vivo. Biochem. Pharmacol..

[53] Oh J., Kim Y., Che L., Kim J.B., Chang G.E., Cheong E., Kang S.-G., Ha Y. (2017). Regulation of cAMP and GSK3 signaling pathways contributes to the neuronal conversion of glioma. PLoS ONE.

[54] Perry J.M., He X.C., Sugimura R., Grindley J.C., Haug J.S., Ding S., Li L. (2011). Cooperation between both Wnt/β-catenin and PTEN/PI3K/Akt signaling promotes primitive hematopoietic stem cell self-renewal and expansion. Genes Dev..

[55] Sato N., Meijer L., Skaltsounis L., Greengard P., Brivanlou A.H. (2004). Maintenance of pluripotency in human and mouse embryonic stem cells through activation of Wnt signaling by a pharmacological GSK-3-specific inhibitor. Nat. Med..

[56] Cai L., Ye Z., Zhou B.Y., Mali P., Zhou C., Cheng L. (2007). Promoting human embryonic stem cell renewal or differentiation by modulating Wnt signal and culture conditions. Cell Res..

[57] Dravid G., Ye Z., Hammond H., Chen G., Pyle A., Donovan P., Yu X., Cheng L. (2005). Defining the role of Wnt/β-catenin signaling in the survival, proliferation, and self-renewal of human embryonic stem cells. Stem Cells.

[58] Davidson K.C., Adams A.M., Goodson J.M., McDonald C.E., Potter J.C., Berndt J.D., Biechele T.L., Taylor R.J., Moon R.T. (2012). Wnt/β-catenin signaling promotes differentiation, not self-renewal, of human embryonic stem cells and is repressed by Oct4. Proc. Natl. Acad. Sci. USA.

[59] Kim H., Wu J., Ye S., Tai C.-I., Zhou X., Yan H., Li P., Pera M., Ying Q.-L. (2013). Modulation of β-catenin function maintains mouse epiblast stem cell and human embryonic stem cell self-renewal. Nat. Commun..

[60] Ying Q.-L., Wray J., Nichols J., Batlle-Morera L., Doble B., Woodgett J., Cohen P., Smith A. (2008). The ground state of embryonic stem cell self-renewal. Nature.

[61] Wray J., Kalkan T., Gomez-Lopez S., Eckardt D., Cook A., Kemler R., Smith A. (2011). Inhibition of glycogen synthase kinase-3 alleviates Tcf3 repression of the pluripotency network and increases embryonic stem cell resistance to differentiation. Nat. Cell Biol..

