# Supplementary material for: Inhibition of GSK3β Promotes Proliferation and Suppresses Apoptosis of Porcine Muscle Satellite Cells
Source: Animals (Basel). 2022 Nov 28;12(23):3328. doi: 10.3390/ani12233328 (PMC9738253; doi:10.3390/ani12233328)
Supplement: Supplementary file 1 [file animals-12-03328-s001.zip › animals-2003878-supplementary.pdf]

Supplementary Information

Inhibition of GSK3β promotes proliferation and suppresses apoptosis of porcine muscle satellite cells

Jinryong Park[1,2], Hyun Woo Choi[3,5] and Kwanseob Shim[4,5,*]

[1] Department of Stem Cell and Regenerative Biotechnology, Konkuk University, Seoul 05029, Korea

[2] 3D Tissue Culture Research Center, Konkuk University, Seoul 05029, Korea

[3] Department of Animal Science, Jeonbuk National University, Jeonju 54896, Korea

[4] Department of Animal Biotechnology, Jeonbuk National University, Jeonju 54896, Korea

[5] Department of Agricultural Convergence Technology, Jeonbuk National University, Jeonju 54896, Korea

[*]Correspondence: ksshim@jbnu.ac.kr (K.S.)

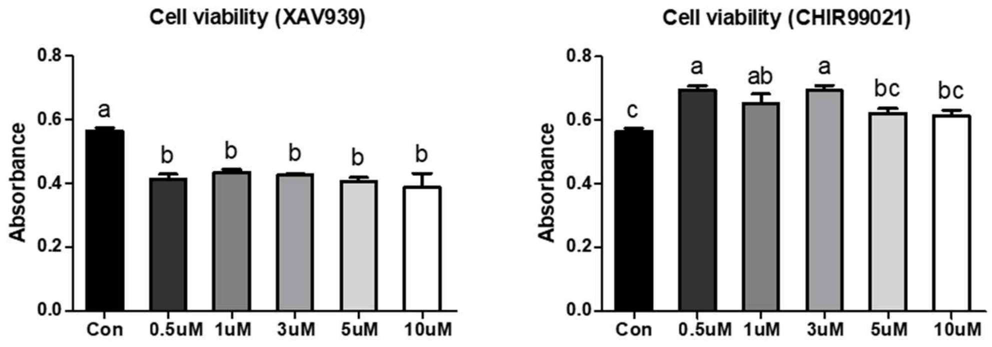

Figure S1. Viability of porcine muscle satellite cells (PMSCs) according to XAV939 or CHIR99021 dose at 48 hours. Values are presented as mean ± SE. [a-c] Different letters represent statistically significant differences among treatment groups ($p < 0.01$).

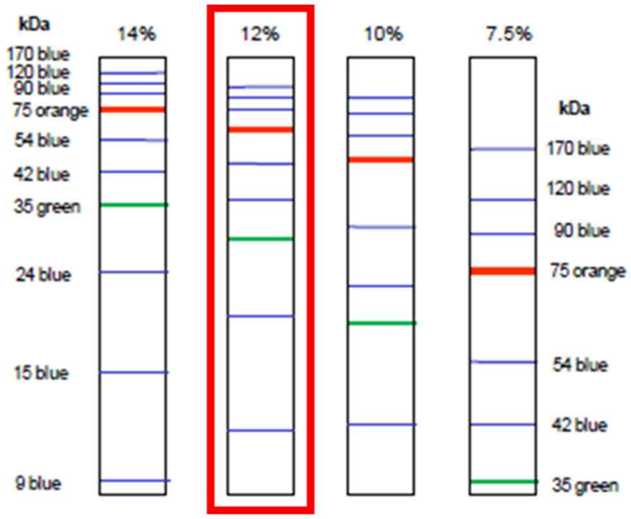

Figure S2. Protein marker information (molecular weight) used for Western blot analysis. Proteins extracted from cells were separated by SDS-PAGE using 12% acrylamide gels, and each protein expression was normalized by GAPDH. Product; Smart Color Protein Marker (Pre-stained), ELPISBIO, EBM-2000.

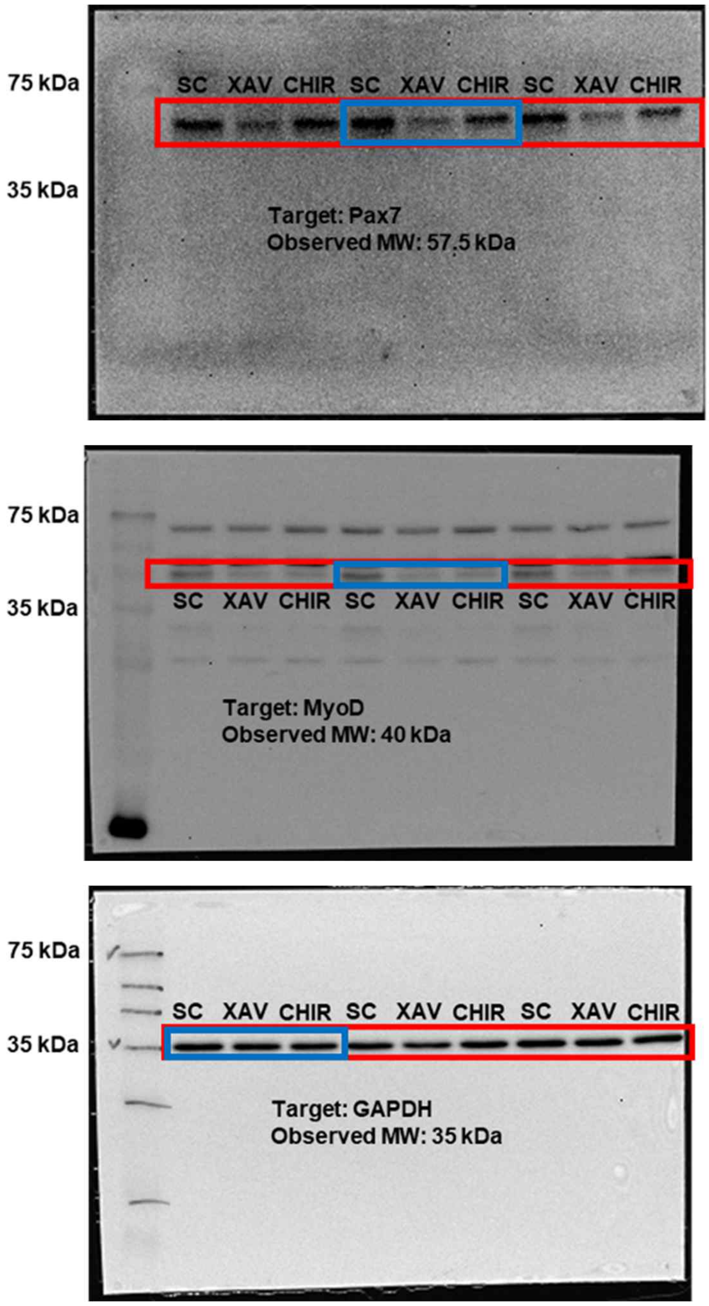

	Pax7/GAPDH	MyoD/GAPDH
SC	0.692218451	0.242795484
XAV	0.273870353	0.052869076
CHIR	0.581537876	0.040899167
SC	0.702694174	0.231571095
XAV	0.267820566	0.053161286
CHIR	0.582979497	0.036319284
SC	0.704525472	0.23256705
XAV	0.274374707	0.052776053
CHIR	0.570515689	0.041506646

Figure S3. Western blotting band of Pax7, MyoD, and GAPDH in PMSCs. The cropped gel image used in the main text is highlighted in blue.

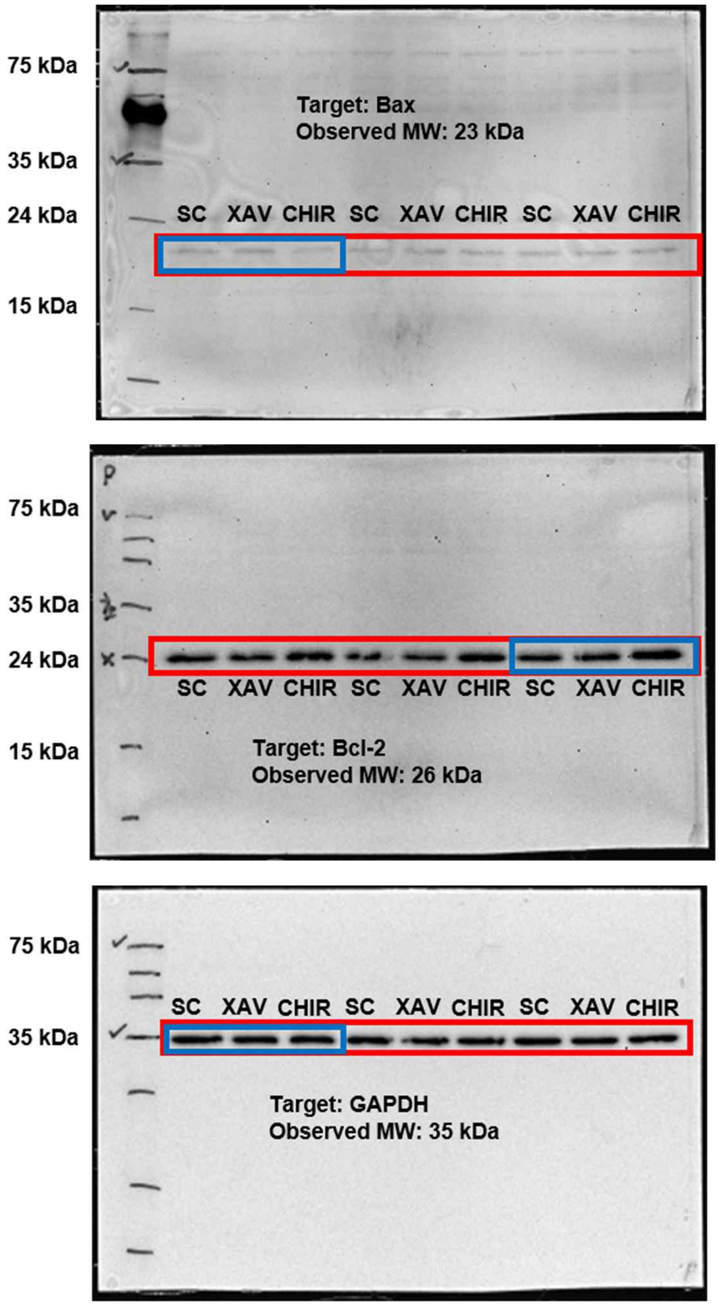

	Bax/GAPDH	Bcl-2/GAPDH
SC	0.169083383	0.754389137
XAV	0.168180247	0.724740247
CHIR	0.11558568	1.05990869
SC	0.161937152	0.741120797
XAV	0.158735514	0.722868139
CHIR	0.10825875	1.118141362
SC	0.158003242	0.756287999
XAV	0.153518218	0.751978469
CHIR	0.104022084	1.043619745

Figure S4. Western blotting band of Bax, Bcl-2, and GAPDH in PMSCs. The cropped gel image used in the main text is highlighted in blue.

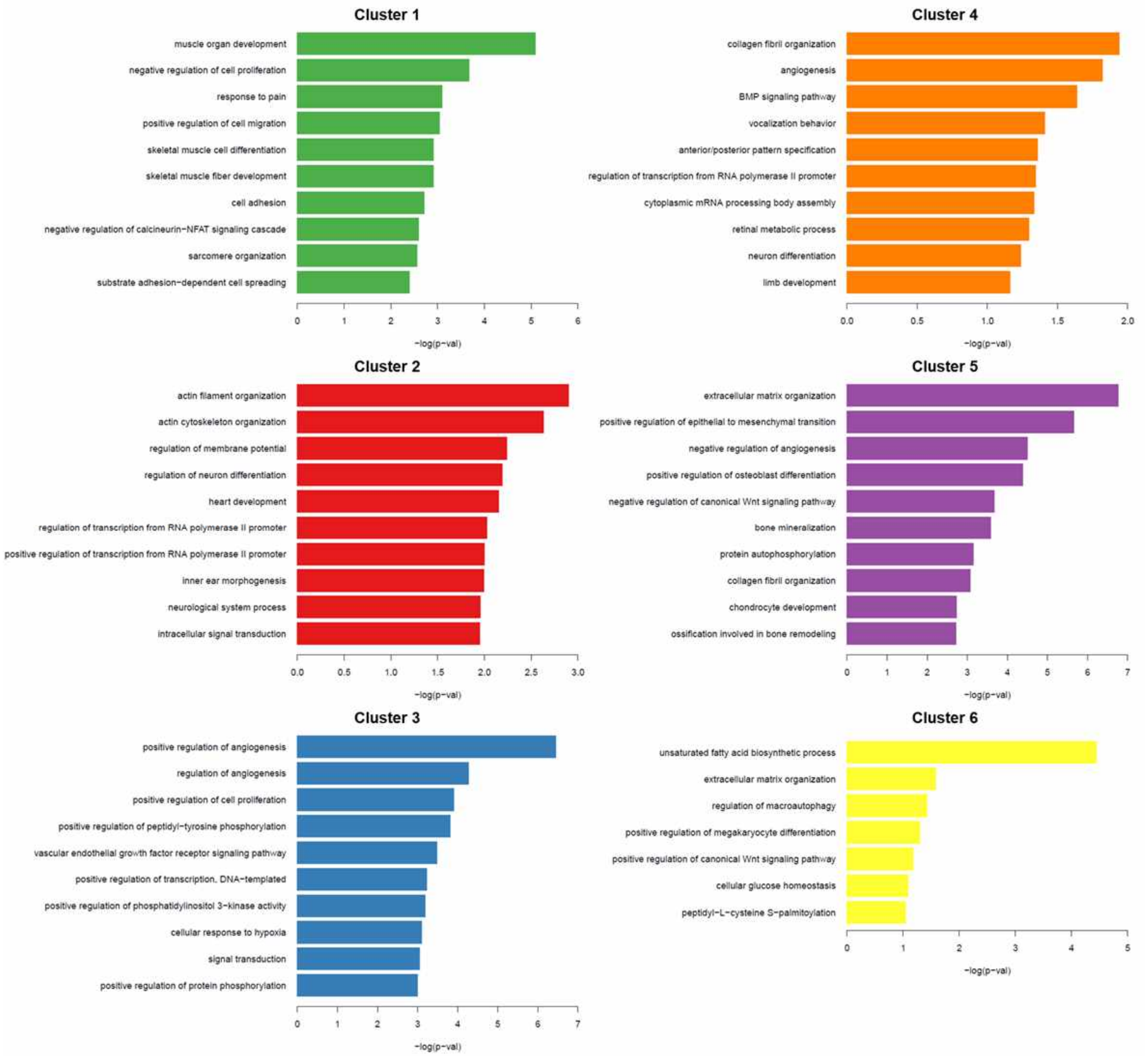

Figure S5. Related terms for each cluster in the heat map analysis.

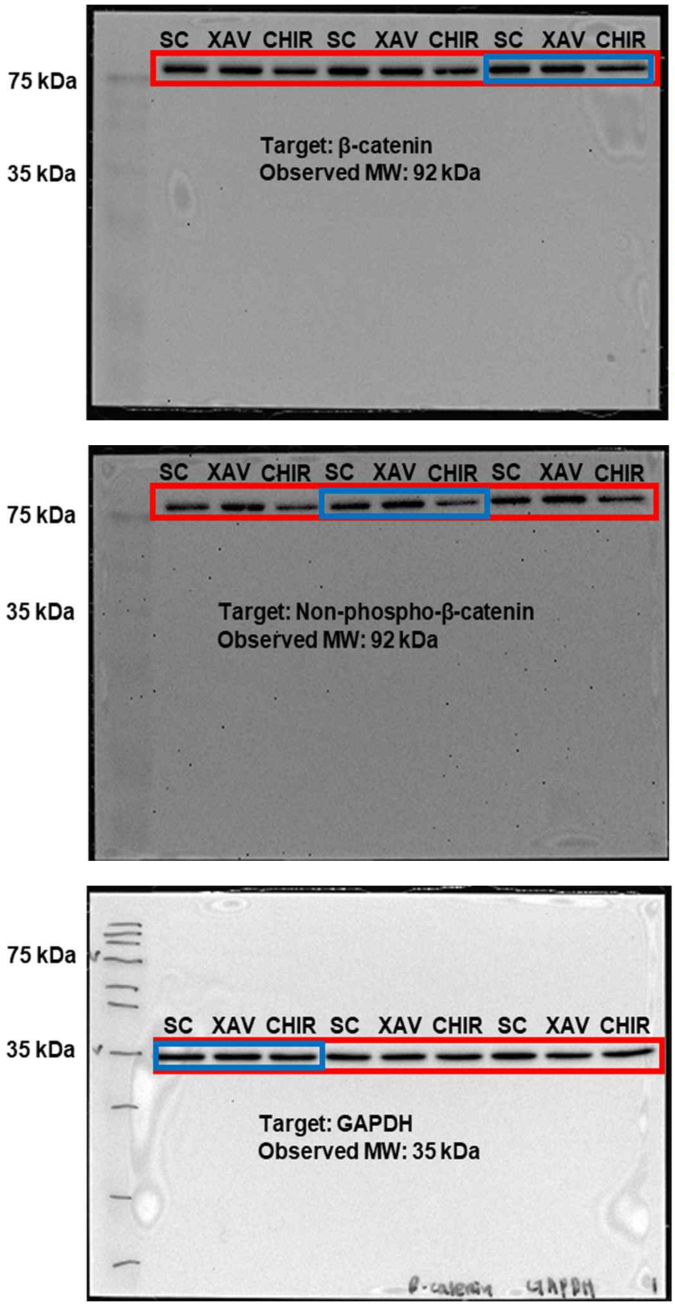

	b-catenin/GAPDH	n-p-b-catenin/GAPDH
SC	1.052216195	0.920126374
XAV	1.046039606	1.109305908
CHIR	0.888669706	0.82213487
SC	1.055644717	0.989848992
XAV	1.029846664	1.16677065
CHIR	0.872817196	0.836810288
SC	1.031940766	1.015761636
XAV	1.065084102	1.18424953
CHIR	0.87496851	0.828776379

Figure S6. Western blotting band of β-catenin, non-phospho-β-catenin, and GAPDH in PMSCs. The cropped gel image used in the main text is highlighted in blue.
